# Effects of Rhizome Extract of *Dioscorea batatas* and Its Active Compound, Allantoin, on the Regulation of Myoblast Differentiation and Mitochondrial Biogenesis in C2C12 Myotubes

**DOI:** 10.3390/molecules23082023

**Published:** 2018-08-13

**Authors:** Junnan Ma, Seok Yong Kang, Xianglong Meng, An Na Kang, Jong Hun Park, Yong-Ki Park, Hyo Won Jung

**Affiliations:** 1 Department of Herbology, College of Korean Medicine, Dongguk University, Gyeongju 38066, Korea; nnzhgn@gmail.com (J.M.); seokppo2@hanmail.net (S.Y.K.); sszywzh@126.com (X.M.); ank4200@gmail.com (A.N.K.); hasangpaul@hanmail.net (J.H.P.); yongki@dongguk.ac.kr (Y.-K.P.); 2 Korean Medicine R&D Center, Dongguk University, Gyeongju 38066, Korea

**Keywords:** allantoin, Chinese yam, C2C12 cells, *Dioscorea batatas*, Dioscoreae Rhizoma, myoblast differentiation, mitochondrial biogenesis

## Abstract

With the aging process, a loss of skeletal muscle mass and dysfunction related to metabolic syndrome is observed in older people. Yams are commonly use in functional foods and medications with various effects. The present study was conducted to investigate the effects of rhizome extract of *Dioscorea batatas* (Dioscoreae Rhizoma, Chinese yam) and its bioactive compound, allantoin, on myoblast differentiation and mitochondrial biogenesis in skeletal muscle cells. Yams were extracted in water and allantoin was analyzed by high performance liquid chromatography (HPLC). The expression of myosin heavy chain (MyHC) and mitochondrial biogenesis-regulating factors, peroxisome proliferator-activated receptor gamma coactivator 1-alpha (PGC-1α), sirtuin-1 (Sirt-1), nuclear respiratory factor-1 (NRF-1) and transcription factor A, mitochondrial (TFAM), and the phosphorylation of AMP-activated protein kinase (AMPK) and acetyl-CoA carboxylase (ACC) were determined in C2C12 myotubes by reverse transcriptase (RT)-polymerase chain reaction (RT-PCR) or western blot. The glucose levels and total ATP contents were measured by glucose consumption, glucose uptake and ATP assays, respectively. Treatment with yam extract (1 mg/mL) and allantoin (0.2 and 0.5 mM) significantly increased MyHC expression compared with non-treated myotubes. Yam extract and allantoin significantly increased the expression of PGC-1α, Sirt-1, NRF-1 and TFAM, as well as the phosphorylation of AMPK and ACC in C2C12 myotubes. Furthermore, yam extract and allantoin significantly increased glucose uptake levels and ATP contents. Finally, HPLC analysis revealed that the yam water extract contained 1.53% of allantoin. Yam extract and allantoin stimulated myoblast differentiation into myotubes and increased energy production through the upregulation of mitochondrial biogenesis regulators. These findings indicate that yam extract and allantoin can help to prevent skeletal muscle dysfunction through the stimulation of the energy metabolism.

## 1. Introduction

Dietary glucose is supplied by meals, and glucose is stored as glycogen in the liver, kidney, and muscle to enable metabolic energy function [1]. Skeletal muscle is largely responsible for regulating carbohydrate metabolism and achieving energy balance in normal feeding [2]. Accordingly, skeletal muscle function deficit, in particular, age-related and disease-related muscle loss, is associated with many chronic diseases including sarcopenia, diabetes and obesity [3]. Such conditions are still difficult to control because causes of muscle loss are multifactorial and influenced by genetics. Recently, there has been increased interest in various functional foods and medicines for promotion of muscle function and maintenance of muscle balance in condition between protein synthesis and degradation [4]. 

The root (Dioscoreae Rhizoma, Chinese yam) of *Dioscorea batatas* Decaisne (=*D. oposita* Thunberg) is a perennial trailing plant of the Dioscoreaceae family. The yam, which is one of the most important herbs in traditional medicine, has long been used as a food and medication with various pharmaceutical functions. In herbology, the yam is neutral in nature, sweet in flavor, and mainly manifests its therapeutic actions in the spleen, lung, and kidney meridians [5,6]. Therefore, yams are utilized to cure yin deficiency in metabolic disorders such as diabetes and hyperthyroidism by tonifying and replenishing qi in meridian organs. Yam is also known to have digestive functions in the stomach and intestines, as well as immune-regulatory and antiaging effects. In modern pharmacology, the yam has been studied for its effects on asthma [7], cancer [8,9,10], diabetes [11,12], and liver damage [13], as well as its anti-oxidation, anti-inflammation, and anti-aging effects [14,15]. The yam contains various compounds such as dioscin [16], steroidal saponins [16,17], saponins, gallic acid, vanillic acid [14], allatoin [12], and protodioscin [18]. In recent years, natural dietary compounds have gained increasing attention as adjuvant therapy due to their relative low toxicity and synergistic effects with current chemotherapeutic agents [19]. 

Allantoin, a diureide of glyoxylic acid, is an active and abundant component of yam [10,12,20]. In vivo studies have shown allantoin to have anti-asthmatic [20], antidiabetic [12,21] and antihypertensive [22] activities, as well as memory-enhancing effects in Alzheimer’s disease [23].

We recently conducted a study that provided scientific evidence of the abilities of various herbs to improve obesity, diabetes and sarcopenia based on clinical practice and found that some herbs have good effects that occur via regulation of the differentiation and mitochondria biogenesis in skeletal muscle [24,25]. Therefore, we investigated the effects of yam water extract and its bioactive compound, allantoin, on myoblast differentiation and mitochondrial biogenesis in C2C12 myotubes. 

## 2. Results

### 2.1. Effects of Yam Extract and Allantoin on Myoblast Differentiation into Myotubes

**Figure 1:**
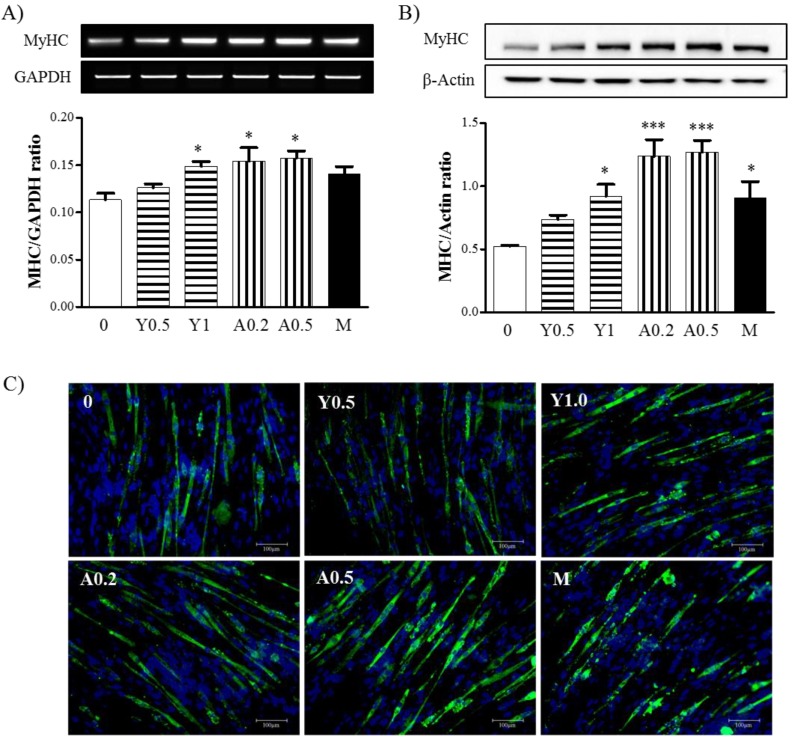
Effects of yam extract and allantoin on the expression of MyHC protein and mRNA in C2C12 myotubes. C2C12 myoblasts were differentiated with DMEM containing 2% HS for 5 days, then treated with or without yam extract (0.5 and 1.0 mg/mL) or allantoin (0.2 and 0.5 mM) for 24 h. Metformin (2.5 mM) was used as a positive control. The expression of MyHC mRNA (**A**) and protein (**B**) was determined by RT-PCR and western blot, respectively. GAPDH and β-actin were used as internal controls. All data were presented as the means ± SEM of three independent experiments. Y, yam extract; A, allantoin; and M, metformin. * *p* < 0.05 and *** *p* < 0.001 vs. non-treated cells; (**C**) the myotubes were stained with anti-MyHC antibody and DAPI, then observed by fluorescence microscopy (original magnification = 200×). Green, MyHC-positive cells; and blue, DAPI-positive nuclei.

To investigate the effects of yam extract and allantoin on myoblast differentiation into myotubes, we determined the expression of Myosin heavy chain (MyHC) mRNA and protein as differentiation markers in C2C12 myotubes using reverse transcriptase (RT)-polymerase chain reaction (RT-PCR) and western blot analysis. Treatment with yam extract (*p* < 0.05 for 1 mg/mL) and allantoin (*p* < 0.001 for 0.2 and 0.5 mM) significantly increased the expression of MyHC mRNA (Figure 1A) and protein (Figure 1B) in C2C12 myotubes compared with non-treated cells as a negative control. We also observed MyHC expression in C2C12 myotubes using immunocytochemistry (Figure 1C), which revealed that MyHC-positive myotubes with an elongated and widened cylinder-shape and multiple nuclei were present in greater numbers in yam extract and allantoin-treated cells than non-treated cells. Metformin-treated cells as a positive control group were also shown to exhibit an increase of MyHC expression, but this was lower than for treatment with yam extract or allantoin. These results indicate that yam extract and allantoin can induce myoblast differentiation into myotubes in skeletal muscle cells. In MTT viability assay, we confirmed that the concentrations of yam extract and allantoin for treatment in C2C12 myoblasts did not affect the viability (data not shown).

### 2.2. Effects of Yam Extract and Allantoin on the Expression of Mitochondria Biogenesis-Regulating Factors in Myotubes

**Figure 2:**
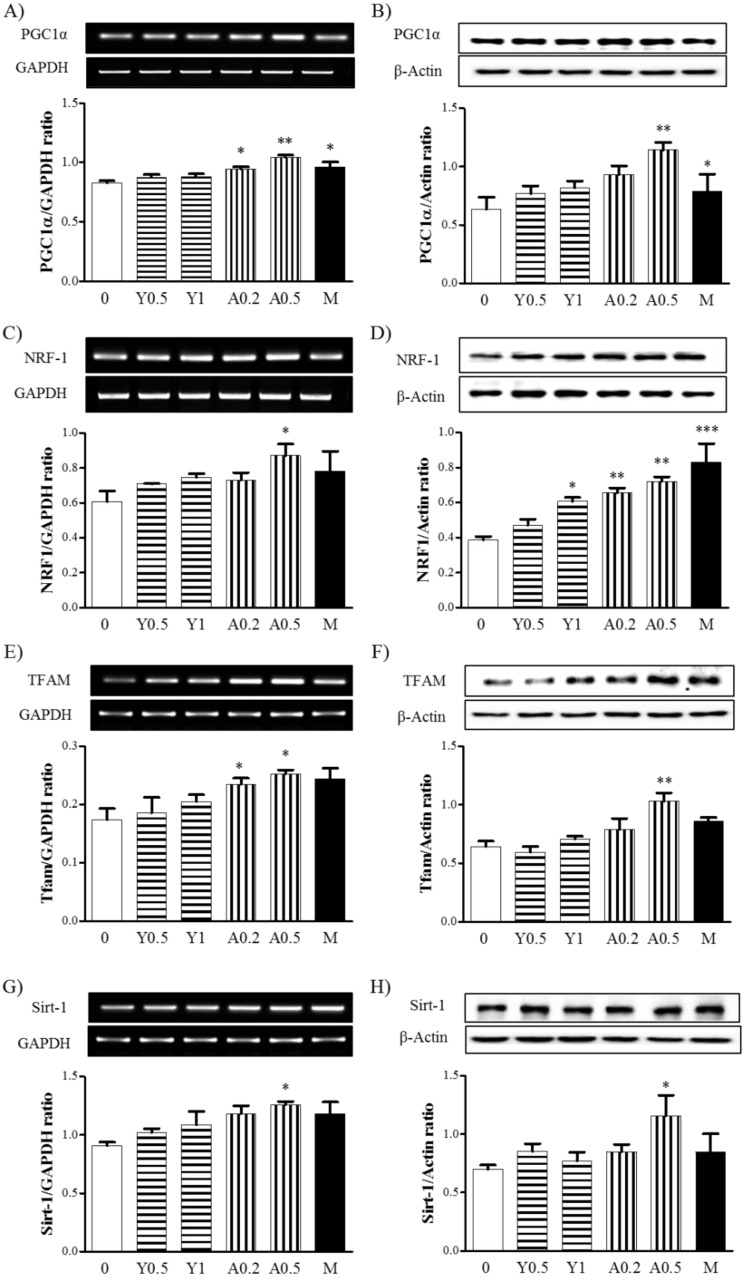
Effects of yam extract and allantoin on the expression of mitochondrial biogenesis-regulating factors in C2C12 myotubes. Differentiated myotubes were treated with or without yam extract (0.5 and 1.0 mg/mL) or allantoin (0.2 and 0.5 mM) for 24 h, after which the expression of PGC1α (**A**,**B**), NRF-1 (**C**,**D**), TFAM (**E**,**F**) and Sirt-1 (**G**,**H**) mRNA (**A**,**C**,**E**,**G**) and protein (**B**,**D**,**F**,**H**) was analyzed by RT-PCR (**A**,**C**,**E**) and western blot (**B**,**D**,**F**), respectively. Metformin (2.5 mM) was used as a positive control. GAPDH and β-actin were used as internal controls. Each band was presented as a representative figure and the histogram was calculated from the band density value of each experiment. All data were presented as the means ± SEM of three independent experiments. Y, yam extract; A, allantoin; and M, metformin. * *p* < 0.05, ** *p* < 0.01 and *** *p* < 0.001 vs. non-treated negative control.

To investigate the effects of yam extract and allantoin on mitochondrial biogenesis in myotubes, we measured the expression of the biogenesis regulating factors, peroxisome proliferator-activated receptor gamma coactivator 1-alpha (PGC-1α), nuclear respiratory factor 1 (NRF-1), transcription factor A, mitochondrial (TFAM) and sirtuin 1 (Sirt-1) mRNA and protein in C2C12 myotubes by RT-PCR and western blot, respectively. Treatment of yam extract (0.5 and 1 mg/mL) and allantoin (0.2 and 0.5 mM) in the myotubes for 24 h increased the expression of PGC1α (Figure 2A,B), NRF-1 (Figure 2C,D), TFAM (Figure 2E,F) and Sirt-1 (Figure 2G,H) mRNA (Figure 2A,C,E,G) and protein (Figure 2B,D,F,H) as compared with non-treated cells. In particular, allantoin in a high concentration (0.5 mM) significantly increased the expression of all regulators of mRNA (*p* < 0.01 for PGC1α, *p* < 0.05 for NRF-1, TFAM and Sirt-1) and protein (*p* < 0.01 for PGC1α, NRF1, and TFAM, *p* < 0.05 for Sirrt1) compared with non-treated cells. Metformin significantly increased the expression of PGC1α (*p* < 0.05 for mRNA and protein) and NRF-1 (*p* < 0.05 for protein) compared with non-treated cells. These results indicate that yam extract and allantoin can enhance mitochondrial biogenesis through upregulation of the expression of the transcription factors.

### 2.3. Effects of Yam Extract and Allantoin on the AMPK and ACC Pathways in Myotubes

**Figure 3:**
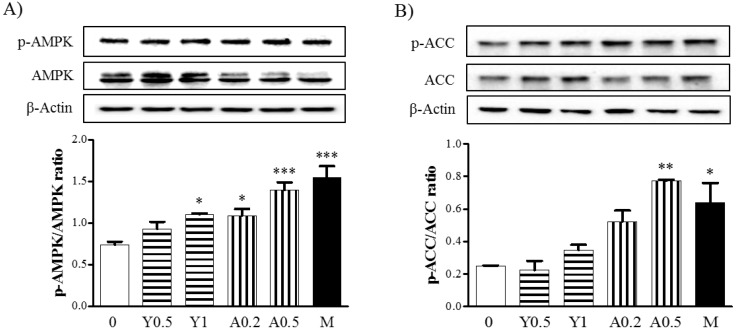
Effects of yam extract and allantoin on the phosphorylation of AMPK and ACC protein inC2C12 myotubes. Differentiated C2C12 myotubes were treated with or without yam extract (0.5 and 1.0 mg/mL) or allantoin (0.2 and 0.5 mM) for 24 h and the phosphorylation of AMPK (**A**) and ACC (**B**) protein was investigated by western blot. Metformin (2.5 mM) was used as a positive control. Each band was presented as a representative figure and a histogram was calculated from the band density value of each experiment. β-actin were used as an internal control. All data were presented as the means ± SEM of three independent experiments. Y, yam extract; A, allantoin; and M, metformin. * *p* < 0.05, ** *p* < 0.01 and *** *p* < 0.001 vs. non-treated negative control.

Next, we investigated the effects of yam extract and allantoin on the signaling pathway activated with mitochondrial biogenesis based on an evaluation of the phosphorylation of AMP-activated protein kinase (AMPK) and Acetyl-CoA carboxylase (ACC) in C2C12 myotubes by western blot. Treatment with yam extract (0.5 and 1 mg/mL) and allantoin (0.2 and 0.5 mM) resulted in the increased phosphorylation of AMPK (Figure 3A) and ACC (Figure 3B) in the myotubes. Moreover, treatment with allantoin at a high concentration (0.5 mM) significantly increased the phosphorylation of AMPK (*p* < 0.001) and ACC (*p* < 0.01) compared with non-treated cells. Metformin as an AMPK activator also significantly increased the phosphorylation of AMPK (*p* < 0.001) and ACC (*p* < 0.05) in myotubes. Metformin significantly increased the phosphorylation of AMPK as AMPK activator (*p* < 0.001) and ACC (*p* < 0.05) compared with non-treated cells. These results indicate that yam extract and allantoin can increase the mitochondrial biogenesis in myotubes through activation of the AMPK/ACC signaling pathway.

### 2.4. Effects of Yam Extract and Allantoin on Glucose Uptake in Myotubes

**Figure 4:**
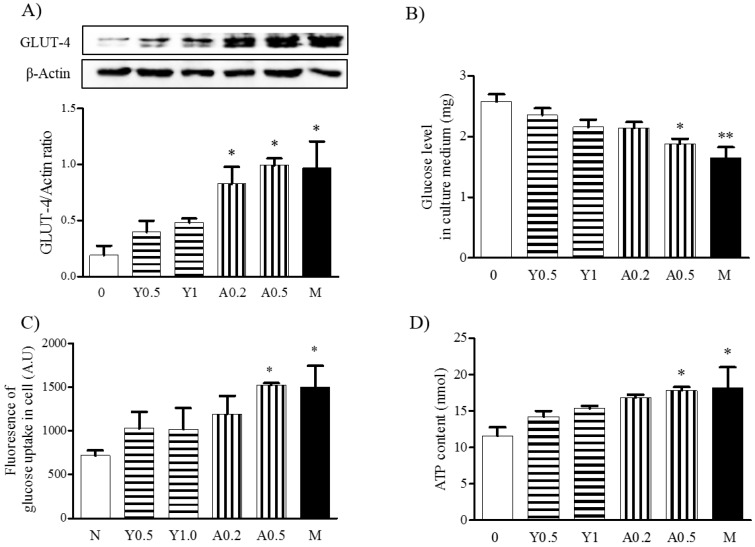
Effects of yam extract and allantoin on the expression of GLUT-4 and the levels of glucose in C2C12 myotubes. Differentiated myotubes were treated with or without yam extract (0.5 and 1 mg/mL) or allantoin (0.2 and 0.5 mM) for 24 h. (**A**) The expression of GLUT-4 protein was determined by western blot. Metformin (2.5 mM) was used as a positive control and β-actin was used as an internal control. Each band was presented as a representative figure and a histogram was calculated from the band density value of each experiment. The levels of glucose in culture medium (**B**) and in the cells (**C**) were measured by a glucose consumption assay and glucose uptake assay, respectively. The contents of ATP in the myotubes were measured using an ATP assay kit (**D**). All data were presented as the means ± SEM of three independent experiments. Y, yam extract; A, allantoin; and M, metformin. * *p* < 0.05 and ** *p* < 0.01 vs. non-treated negative control.

To investigate the effects of yam extract and allantoin on glucose uptake into myotubes, we evaluated the expression of Glucose transporter type 4 (GLUT-4) protein and measured the glucose levels in culture medium and in cells using western blot, glucose consumption assay, and glucose uptake assay, respectively. The results revealed that the expression of GLUT-4 in the myotubes was significantly increased by treatment with yam extract (0.5 and 1 mg/mL) and allantoin (0.2 and 0.5 mM) in a dose-dependent manner. Moreover, allantoin treatment induced a significant increase in GLUT-4 expression (*p* < 0.05 for 0.2 and 0.5 mM), which was more effective than yam extract treatment (Figure 4A). Additionally, glucose was significantly decreased in culture medium of allantoin-treated myotubes (*p* < 0.05 for 0.2 and 0.5 mM, Figure 4B), while the cellular levels were significantly increased (*p* < 0.05 for 0.5 mM, Figure 4C). Metformin treatment also significantly decreased glucose levels in culture medium (*p* < 0.01) and significantly increased cellular glucose levels (*p* < 0.001) compared with non-treated cells. Metformin significantly increased GLUT-4 expression (*p* < 0.05) and glucose uptake (*p* < 0.05) in myotubes compared with non-treated cells. These results indicate that yam extract and allantoin can stimulate glucose uptake in myotubes by increasing the GLUT-4 expression.

### 2.5. Effects of Yam Extract and Allantoin on ATP Production in Myotubes

To investigate the effects of yam extract and allantoin on energy production in myotubes, we measured the ATP contents in myotubes. As shown in Figure 4D, the treatment of myotubes with yam extract and allantoin led to dose-dependent increases in ATP production, with significantly increased ATP levels being observed in response to allantoin (*p* < 0.05 for 0.5 mM). Metformin significantly increased ATP production (*p* < 0.05) in myotubes compared with non-treated cells. These results indicate that yam extract and allantoin can enhance the energy production in myotubes, which might be related to the upregulation of the mitochondrial biogenesis-regulating factors, as shown in Figure 2. 

### 2.6. HPLC Analysis

**Figure 5:**
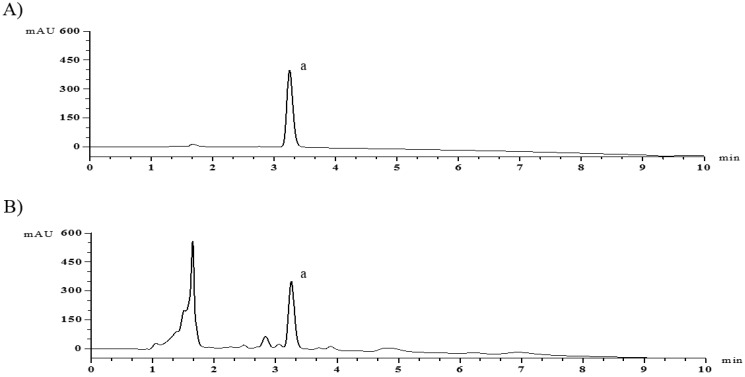
HPLC analysis of allantoin in the yam extract: (**A**) allantoin as a standard compound, and (**B**) allantoin in the water extract. a, allantoin (retention time: 3.253 min).

To analyze the content of allantoin in yam extract, we conducted HPLC analysis and then compared the retention time of samples with that of authentic standard (Figure 5A). The content of allantoin in yam water extract was subsequently calculated by comparison of peak areas (Figure 5B). The equation of the calibration curves for allantoin was *y* = 16039*x* − 16.79. In addition, the correlation coefficient of the calibration curve was higher than 0.9995, and the concentration of allantoin in the extract was 1.53%. The relative standard deviations of precision and repeatability were 0.67% and 1.85%, respectively.

## 3. Discussion

Social, health, and technological developments have resulted in increases in the proportion of older people increasing worldwide along with increasing life expectancy [25]. The aging process is responsible for many changes in body composition, particularly a loss of skeletal muscle mass. Muscle mass loss and dysfunction in older people are associated with various types of disease, injury or aging, which significantly increases the cost of health care [26]. Age-related reductions in muscle mass known as sarcopenia induce negative effects on muscle strength and muscle quality, as well as decreased physical function, all of which lead to mobility impairment, disability, fatigue, risk of metabolic disorders, falls, and mortality in older adults [2]. Recent research strategies have focused on factors associated with muscle mass and strength, as well as nutritional interventions; specifically, diets rich in proteins and antioxidant supplements and various exercise-related interventions are thought to increase muscle strength and physical function [25,27]. Although potent pharmaceutical treatments such as hormone therapies, angiotensin converting enzyme inhibitors and ghrelin agonists have been studied, there has been little convincing evidence of their effects or they have induced adverse side effects [27]. Nevertheless, it is necessary to find and implement interventions to prevent and treat sarcopenia in the ageing population.

Dietary supplemental herbs with many beneficial effects have long been considered to enhance health status and physical strength as well as to improve abnormal status among the elderly [28]. Yams are commonly used in medications because of their various pharmaceutical functions, which include the enhancement of the digestive process in the stomach and intestines, immune regulation, and antiaging, antiinflammation and antioxidation effects. In traditional medicine, yams are known as a nourishing herb that alleviates yin deficiency in the spleen, lung, and kidney by providing a supplementary energy (qi), therefore, it is used to treat metabolic syndromes such as obesity, diabetes, and hypothyroidism [29]. In addition, yams have been used to prevent the aging process (e.g., muscle weakness) because they control muscle function by spleen control [30]. However, there is little known about the medicinal effects of yams on muscle function. In the present study, we investigated whether yam extract and its active compound, allantoin, could help enhance the muscle function in myotubes. The results revealed that yam extract and allantoin significantly increased myoblast differentiation into myotubes in C2C12 cells and mitochondrial biogenesis through the upregulation of the mitochondrial transcription factors, PGC1α, TFAM, NRF-1, and Sirt-1 via activation of the AMPK/ACC signaling pathway. 

To overcome muscle wasting in sarcopenia, it is necessary to stimulate the myogenesis pathway or inhibit the muscle wasting process. Satellite cells such as C2C12 myoblasts undergo expansion and migration and differentiate into multinucleated fibers, myotubes via myoblasts fusion [31]. Myoblast differentiation is orchestrated by myogenic regulatory factors (MRFs) such as myoblast determination protein (MyoD), MRF4, myogenic factor 5 (Myf5) and myogenin [31]. Mature myotubes also express structural muscle proteins such as tropomyosin and MyHC, which is the motor protein of muscle thick filaments and a specific mature marker protein [32]. In the present study, the treatment of yam extract and allantoin significantly increased the expression of MyHC mRNA and protein in C2C12 myotubes, which was higher than Metformin-treatment, suggesting that yams and allantoin can facilitate myoblast differentiation in muscle cells; however, future investigations of the regulation of MRFs and their signals are still needed to better understand the effects of yams and allantoin on myogenesis. Meanwhile, in MTT assay, allantoin did not decrease the viability up to 1 mM in C2C12 myotubes, but its treatment showed a lower expression of MyHC than treatment with 0.5 mM (data not shown). Therefore, we used concentrations of allantoin at 0.2 and 0.5 mM for this study. 

Skeletal muscle, which is a key tissue involved in the control of the energy metabolism, processes up to 75% of insulin-stimulated glucose disposal by the translocation of GLUT4 to the plasma membrane in response to the activation, or resulting in the activation, of the AMP-activated protein kinase (AMPK) pathway [33]. AMPK is a key energy sensor controlling metabolic homeostasis at both the cellular and whole-body levels and is involved in many other cellular processes including cell cycle regulation and endothelial and vascular relaxation [34]. Therefore, it has been considered a subject in recent studies of metabolic syndrome such as obesity, insulin resistance, dyslipidemia, and diabetes mellitus [34,35]. In myoblast differentiation, cellular ATP consumption elevates the cellular AMP/ATP ratio which stimulates ATP generation through AMPK activation [35,36]. AMPK activation mediates the increased expression of GLUT-4 and mitochondrial biogenesis and regulates fatty acid oxidation via the phosphorylation of ACC and the expression of Sirt-1 [37]. Sirt-1 is another downstream regulator of the glucose and lipid metabolism that is known to improve insulin sensitivity and to stimulate mitochondrial biogenesis in skeletal muscle via interaction with AMPK/PGC1α [38]. PGC1α is a major regulator of mitochondrial biogenesis that activates the expression of its downstream transcription factors, NRF-1and TFAM [39]. In the present study, yam extract (0.5 and 1 mg/mL) and allantoin (0.2 and 0.5 mM) increased the expression of PGC1α, Sirt-1, NRF-1, and TFAM in C2C12 myotubes. These results indicate that the increasing levels of glucose uptake and ATP in the myotubes are connected with the upregulation of the mitochondrial biogenesis regulating factors PGC1α, Sirt-1, NRF-1, and TFAM, as well as with activation of the AMPK/ACC pathway. Therefore, yam extract and allantoin could help to elevate energy production by increasing mitochondrial biogenesis in skeletal muscle. Recently, it was reported that metformin increases mitochondrial energy formation in L6 muscle cells [40]. We investigated the effects of yam extract and allantoin on the expression of mitochondrial biogenesis regulating factors, PGC1α, Sirt-1, NRF-1, and TFAM, at one-time treatment point; however, these should be considered at multiple time points to observe any changes in mitochondrial biogenesis.

In *Dioscorea* species, the rhizomes of *D. batatas*, *D. opposite*, and *D. japonica* are commonly used as cultivated edible yams, but many wild varieties have rhizomes with different tastes and are not generally edible. However, it was reported that batatasin IV, raspberry ketone, 2-methoxy-4′-hydroxyacetophenone, (3*R*,5*S*)-3,5-dihydroxy-1,7-bis(4-hydroxy-3-methoxyphenyl) heptane, β-sitosterol, blumenol A, dihydropinosylvin, stilbostemin N, butyl-β-d-fructofuranoside, allantoin, dioscin, and coreajaponins A(1) and B(2) were found in 50% EtOH extract of *D. japonica* [41], and steroidal saponins such as protodioscin, dioscin, and gracilin were found in CH_3_CN extract of in *D. tokoro* (wild yam) [18], and batatasin I and 6,7-dihydroxy-2,4-dimethoxy phenanthrene were found in MeOH extract of *D. batatas* aerial bulbil [42]. Allantoin was identified in the water extract of flesh and peel of *D. opposite* by HPLC-PAD [9], and of the tuber and bulbil of *D. batatas* by HPLC [43]. In our analysis, allantoin (0.36 mg/mL, 1.53%) was found in the water extract of *D. batatas* rhizome. Meanwhile, we detected additional small peak beside to allantoin in HPLC analysis. Recently, it was reported that the adenosine is detected at similar retention time with allantoin because of similar polar and structure [44], however, it will be necessary to analysis.

We used metformin as a reference drug to compare the efficacy of yam extract and allantoin on mitochondrial biogenesis. Metformin treatment to C2C12 myotubes significantly increased the glucose uptake and ATP levels with upregulation of PGC1α and NRF-1 expression, and phosphorylation of AMPK, but these effects were seen to be lower than in allantoin. Thus, the antidiabetic effects of allantoin have been reported in streptozotocin-induced diabetic rats through modulating lipid profiling and increasing GLP-1 release [12] and glucose uptake with GLUT-4 expression in skeletal muscle [20,45]. Our results also suggest that allantoin has an improvement potential for muscle dysfunction in disease conditions through increasing energy formation in muscle. Under diabetic conditions including insulin resistance, hyperglycemia is a risk factor for age-related loss of muscle mass in sarcopenia, which induces muscle synthesis reduction, chronic inflammation, and mitochondrial dysfunction [46]. Therefore, in our further study, the effects of yam extract and allantoin will be investigated in skeletal muscle dysfunction in type 2 diabetes mouse models which show insulin resistance and impaired mitochondrial function in muscle.

## 4. Materials and Methods

### 4.1. Materials

Allantoin and metformin were purchased from Sigma-Aldrich (St. Louis, MO, USA). DMEM and penicillin/streptomycin solution were acquired from Corning (Corning, NY, USA). Fetal bovine serum (FBS), horse serum (HS) and penicillin/streptomycin (P/S) solution were obtained from Merck Millipore (Temecula, CA, USA). An ATP colorimetric assay kit was procured from BioVision Inc. (Milpitas, CA, USA). Anti-Sirt-1, TFAM, NRF-1, AMPK, phospho-AMPK, total-AMPK, phospho-ACC, and total-ACC antibodies were purchased from Cell Signaling Technology (Danvers, MA, USA). Anti-MyHC and GLUT4 antibodies were acquired from Santa Cruz Biotechnology (Dallas, TX, USA). Anti-PGC1α antibody and radioimmunoprecipitation assay (RIPA) buffer were obtained from Thermo Fisher Scientific (Waltham, MA, USA).

### 4.2. Preparation of Yam Extract

Dried rhizome of *D. batatas* was purchased from an herbal company (Kwangmyungdang, Ulsan, Korea) and identified by Professor Y.-K. Park, a medical botanist in herbology at College of Korean Medicine, Dongguk University (DUCOM). A voucher specimen was deposited at the herbarium of DUCOM (2017DR). Yams (200 g) were extracted by boiling in2 L of water for 3 h, filtered through Whatman Grade 1 filter paper (Sigma-Aldrich, St Louis, MO, USA), concentrated under a vacuum rotary evaporator (Eyela. Co., Ltd., Tokyo, Japan) at 60 °C, and then lyophilized in a freeze-dryer (IlShinBioBase Co., Yangju, Korea) at −80 °C under 5 mTorr. Yam extract (yield = 11.4%) was stored at 4 °C, dissolved in 1× Phosphate Buffer solution (PBS), and filtered through a syringe filter (0.45 μm, Corning, Wiesbaden, Germany) before being used in in vitro experiments. 

### 4.3. Cell Culture and Drug Treatments

C2C12 myoblasts, a mouse skeletal muscle line, were purchased from the American Type Culture Collection (ATCC; Manassas, VA, USA) and grown in DMEM supplemented with 10% (*v*/*v*) FBS and1% P/S solution in a humidified atmosphere of 95% air and 5% CO_2_ at 37 °C. At 85–95% confluence, myoblasts were induced to differentiate in DMEM with 2% HS once every day for an additional 5 days. The C2C12 myotubes were then treated with or without different concentrations of yam extract or allantoin. Metformin (2.5 mM) was used as a positive control drug. Allantoin and metformin were dissolved in 1× PBS (pH 7.4). 

### 4.4. Western Blot 

After cells were lysed in ice-cold RIPA buffer containing a phosphatase inhibitor cocktail (Thermo Fisher Scientific), lysates were centrifuged at 12,000× *g* for 20 min at 4 °C. Protein concentrations of the lysates were then quantified using the protein assay solution (BioRad, Hercules, CA, USA). Next, 50 μg of protein was separated by SDS-polyacrylamide gel electrophoresis (PAGE) and transferred onto nitrocellulose membrane. The membrane was then blocked with 5% skim milk for 1 h at room temperature, after which it was immunoblotted with primary antibodies against MyHC, PGC1α, NRF-1, TFAM, Sirt-1, AMPK (total or phosphor-forms), and ACC (total or phosphor forms), as well as β-actin as an internal control overnight at 4 °C. Following immunoblotting, the membranes were washed three times with 1× tris-buffered saline (pH 7.4) containing 0.1% tween-20 (TBST) buffer, then reacted with horseradish peroxidase (HRP)-labeled anti-mouse or anti-rabbit IgG. All immunoblots were subsequently washed with 1× TBST three times, then developed using ECL^TM^ Western blotting detection reagent (GE Healthcare, Pittsburgh, PA, USA). Finally, bands were detected using a ChemiDoc MP Imaging System (BioRad, Hercules, CA, USA) and quantified by densitometry using the Image J programing software (1.51p 22 for Windows, NIH, Bethesda, MD, USA).

### 4.5. Reverse Transcriptase (RT)-Polymerase Chain Reaction (RT-PCR)

Total RNA was isolated from cells by TRIzol reagent (GibcoBRL Life Technologies Inc., Grand Island, NY, USA) according to the manufacturer’s instructions. The RNA concentration was then quantified using a Nano Drop ND-1000 spectrophotometer (NanoDrop Technologies, Inc., Wilmington, DE, USA). Next, cDNA was generated from 1 µg of total RNA using a Reverse Transcription System kit (Promega, Fitchburg, WI, USA), after which RT-PCR was conducted using a Blend Taq PCR kit (Toyobo, Osaka, Japan) and primers specific to the target genes (Table 1). For PCR, the samples were subjected to pre-denaturation at 94 °C for 2 min, followed by 30 cycles of denaturation for 30 s at 94 °C, annealing for 30 s at 56–60 °C, and extension for 1 min at 72 °C. Finally, the bands were detected with the BioRad ChemiDoc MP imaging system and quantified by densitometry using the Image J programming software.

### 4.6. Immunocytochemistry

Differentiated myotubes were seeded on Thermanox plastic cover slips (Nunc^TM^, Thermo Fisher Scientific) and differentiated using a common culture method for 5 days. Samples on cover slips were then fixed with 4% paraformaldehyde for 10 min, after which they were permeabilized with 0.1% Triton X-100 (Sigma-Aldrich) for 20 min. After washing with 1× PBS, cover slips were blocked with 5% bovine serum albumin (BSA) for 30 min at room temperature, then incubated with anti-MyHC antibody overnight at 4 °C. Cover slips were subsequently labelled with AlexaFluor 488-conjugated goat anti-rabbit antibody for 1 h at room temperature, then counterstained with DAPI for 5 min. Finally, the expression of MyHC was observed using a fluorescence microscope (Leica DM2500, Leica microsystems, Wetzlar, Germany).

### 4.7. Glucose Assay 

Glucose consumption was determined in culture media using a glucose assay kit (Sigma-Aldrich, St. Louis, MO, USA). Briefly, cell culture supernatants were harvested and diluted with deionized water, after which 50 μL of the diluted sample was mixed with an equal volume of assay buffer including *o*-dianisidine in a 96-well plate. The mixture was then incubated at 37 °C for 30 min, at which time the reaction was stopped by adding 50 μL of H_2_SO_4_ and the absorbance (O.D.) at 540 nm was measured in a microtiter reader (UVM340, Asys Hitech Gmbh, Eugendorf, Austria). The glucose consumption in each sample was calculated using a calculation formula from a standard curve. 

Next, the cellular levels of glucose were measured in C2C12 myotubes using a glucose uptake cell-based assay kit (Cayman Chemical Co., Ann Arbor, MI, USA). Briefly, C2C12 myotubes were treated with or without yam extract and allantoin at different concentrations in glucose-free medium containing 100 µg/mL of 2-[*N*-(7-nitrobenz-2-oxa-1,3-diazol-4-yl) amino]-2-deoxy-d-glucose (NBDG) for 4 h. After harvesting the cells, cell-based assay buffer (200 µL) was added to each well. The amount of 2-NBDG taken up by the myotubes was then measured with fluorescent filters that detected fluorescein (excitation/emission = 485/650) using a Glomax multi detection system (Promega Biosystems, Sunnyvale, CA, USA). 

### 4.8. ATP Assay 

Total ATP contents were determined using an ATP colorimetric assay kit (BioVision) according to the manufacturer’s instructions. Briefly, C2C12 myotubes were harvested and homogenized in 100 μL ATP assay buffer, after which 50 μL of deproteinized cell lysate was mixed with 50 μL of reaction mix containing ATP probe, ATP converter, and developer in a 96-well plate. The mixture was subsequently incubated at room temperature for 30 min, at which time the absorbance (O.D.) at 570 nm was measured using a microtiter reader. Finally, the concentration of ATP (μM) in each sample was calculated using a calculation formula generated from a standard curve. 

### 4.9. HPLC Analysis

To identify allantoin in yam extract, HPLC was conducted using an Agilent 1260 infinity II quaternary system equipped with a G7129A vial sampler and a WR G7115 Adiode array detector (Agilent, Waldbronn, Germany) and a ZORBAXNH2 column (4.6 × 150 mm, 5-micron). Chromatographic separation was performed using a gradient solvent system consisting of acetonitrile (HPLC grade, Merck, Darmstadt, Germany) (B) and water (HPLC grade, Merck) (A). The gradient program was as follows: 0 min, 25% B; 5 min, 17% B; 10 min, 17% B. The injection volume was 10 μL and the column eluent was monitored at UV 200 nm while chromatography was performed at 30 °C with a flowrate of 1.0 mL/min. The HPLC pattern of allantoin in yam water extract has been reported in the literature [9,26].

### 4.10. Statistical Analysis

The data are presented as means ± standard errors of means (SEMs) of three independent experiments. Differences between groups were identified by the Student’s *t*-test using the GraphPad Prism program (ver. 5.0, GraphPad Software, La Jalla, CA, USA) and *p*-values < 0.05 were considered statistically significant.

## 5. Conclusions

Yam water extract and its active compound, allantoin, significantly improved C2C12 myoblast differentiation into myotubes by increasing the MyHC expression. In addition, these compounds significantly increased the glucose uptake and ATP production in myotubes through the upregulation of the mitochondrial biogenesis-regulating factors PGC1α, NRF-1, TFAM, and Sirt-1 and activation of the AMPK/ACC signaling pathway. In particular, the effects of allantoin on biogenesis were shown to be more pronounced than metformin. Our results suggest that yam and allantoin can help prevent energy loss in muscle dysfunction and are applicable for use as natural sources for food materials and medication for the prevention of sarcopenia and treatment.

## Figures and Tables

**Table 1:** Primer sequences of the target genes for PCR.

Primers		Accession No.	Sequence (5′→3′)
MyHC	Forward	NM 001039545.2	TGA ACT GGA GGG TGA GGT AG
	Reverse	NM 001039545.2	TTC GGT CTT CTT CTG TCT GG
PGC1α	Forward	XM 006503779.3	CAC CAA ACC CAC AGA AAA CAG
	Reverse	XM 006503779.3	GGG TCA GAG GAA GAG ATA AAG TTG
NRF-1	Forward	XM 017321445.1	ACC CTC AGT CTC ACG ACT AT
	Reverse	XM 017321445.1	GAA CAC TCC TCA GAC CCT TAA C
TFAM	Forward	XM 017313918.1	CAC CCA GAT GCA AAA CTT TCA G
	Reverse	XM 017313918.1	CTG CTC TTT ATA CTT GCT CAC AG
Sirt-1	Forward	NM 001159589.2	GAT CCT TCA GTG TCA TGG TT
	Reverse	NM 001159589.2	GAA GAC AAT CTC TGG CTT CA
Gapdh	Forward	XM_017321385.1	CAG CCT CGT CCC GTA GAC A
	Reverse	XM_017321385.1	CGC TCC TGG AAG ATG GTG AT

## Abbreviations

ACC	Acetyl-CoA carboxylase
AMPK	AMP-activated protein kinase
BSA	Bovine serum albumin
DMEM	Dulbecco’s modified Eagle’s medium
FBS	Fetal bovine serum
HRP	Horseradish peroxidase
HS	Horse serum
MRFs	Myogenic regulatory factors
Myf5	Myogenic factor 5
MyoD	Myoblast determination protein
MyHC	Myosin heavy chain
NRF-1	Nuclear respiratory factor-1
RT-PCR	Reverse transcriptase-polymerase chain reaction
PAGE	Polyacrylamide gel electrophoresis
PBS	Phosphate buffered saline
PGC1α	Peroxisome proliferator-activated receptor gamma coactivator
RIPA	Radioimmunoprecipitation assay
Sirt-1	Sirtuin 1
TBST	Tris-buffered saline containing 0.1% tween-20
TFAM	Transcription factor A, mitochondrial
HPLC	High Performance Liquid Chromatography

## References

[1] Pyla R., Pichavaram P., Fairaq A., Park M.A., Kozak M., Kamath V., Patel V.S., Segar L. (2015). Altered energy state reversibly controls smooth muscle contractile function in human saphenous vein during acute hypoxia-reoxygenation: Role of glycogen, AMP-activated protein kinase, and insulin-independent glucose uptake. Biochem. Pharmacol..

[2] Papa E.V., Dong X., Hassan M. (2017). Skeletal Muscle Function Deficits in the Elderly: Current Perspectives on Resistance Training. J. Nat. Sci..

[3] Kalyani P.R., Corriere M., Ferrucci L. (2014). Age-related and disease-related muscle loss: The effect of diabetes, obesity, and other diseases. Lancet Diabetes Endocrinol..

[4] Jung H.W., Kang A.N., Kang S.Y., Park Y.K., Song M.Y. (2017). The Root Extract of Puerarialobata and Its Main Compound, Puerarin, Prevent Obesity by Increasing the Energy Metabolism in Skeletal Muscle. Nutrients.

[5] Kim I.R., Kim H.C., Kook Y.B., Park S.J., Park Y.K., Park J.H., Seo B.I., Seo Y.B., Song H.J., Shin M.K. (2007). Herbology.

[6] Ling Y.Q. (2000). Chinese Herbal Medicine.

[7] Park H.S., Kim M.J., Moon H.B. (1994). Occupational asthma caused by two herb materials, *Dioscorea batatas* and *Pinellia ternata*. Clin. Exp. Allergy.

[8] Miyoshi N., Nagasawa T., Mabuchi R., Yasui Y., Wakabayashi K., Tanaka T., Ohshima H. (2011). Chemoprevention of azoxymethane/dextran sodium sulfate-induced mouse colon carcinogenesis by freeze-dried yam sanyaku and its constituent diosgenin. Cancer Prev. Res..

[9] Liu Y., Li H., Fan Y., Man S., Liu Z., Gao W., Wang T. (2016). Antioxidant and Antitumor Activities of the Extracts from Chinese Yam (*Dioscorea* opposite Thunb) Flesh and Peel and the Effective Compounds. J. Food Sci..

[10] Tsukayama I., Toda K., Takeda Y., Mega T., Tanaka M., Kawakami Y., Takahashi Y., Kimoto M., Yamamoto K., Miki Y. (2018). Preventive effect of *Dioscorea japonica* on squamous cell carcinoma of mouse skin involving down-regulation of prostaglandin E2 synthetic pathway. J. Clin. Biochem. Nutr..

[11] Kim S., Jwa H., Yanagawa Y., Park T. (2012). Extract from *Dioscorea batatas* ameliorates insulin resistance in mice fed a high-fat diet. J. Med. Food.

[12] Go H.K., Rahman M.M., Kim G.B., Na C.S., Song C.H., Kim J.S., Kim S.J., Kang H.S. (2015). Antidiabetic Effects of Yam (*Dioscorea batatas*) and Its Active Constituent, Allantoin, in a Rat Model of Streptozotocin-Induced Diabetes. Nutrients.

[13] Yeh Y.H., Hsieh Y.L., Lee Y.T. (2013). Effects of yam peel extract against carbon tetrachloride-induced hepatotoxicity in rats. J. Agric. Food Chem..

[14] Chiu C.S., Deng J.S., Chang H.Y., Chen Y.C., Lee M.M., Hou W.C., Lee C.Y., Huang S.S., Huang G.J. (2013). Antioxidant and anti-inflammatory properties of Taiwanese yam (*Dioscorea japonica* Thunb. var. *pseudojaponica* (Hayata) Yamam.) and its reference compounds. Food Chem..

[15] Cronin H., Draelos Z.D. (2010). Top 10 botanical ingredients in 2010 anti-aging creams. J. Cosmet. Dermatol..

[16] Jeon J.R., Lee J.S., Lee C.H., Kim J.Y., Kim S.D., Nam D.H. (2006). Effect of ethanol extract of dried Chinese yam (*Dioscorea batatas*) flour containing dioscin on gastrointestinal function in rat model. Arch. Pharm. Res..

[17] Aumsuwan P., Khan S.I., Khan I.A., Ali Z., Avula B., Walker L.A., Shariat-Madar Z., Helferich W.G., Katzenellenbogen B.S., Dasmahapatra A.K. (2016). The anticancer potential of steroidal saponin, dioscin, isolated from wild yam (*Dioscorea villosa*) root extract in invasive human breast cancer cell line MDA-MB-231 in vitro. Arch. Biochem. Biophys..

[18] Oyama M., Tokiwano T., Kawaii S., Yoshida Y., Mizuno K., Oh K., Yoshizawa Y. (2017). Protodioscin, Isolated from the Rhizome of *Dioscorea* tokoro Collected in Northern Japan is the Major Antiproliferative Compound to HL-60 Leukemic Cells. Curr. Bioact. Compd..

[19] Pan P., Chad S., Jian H.Y., Hui Z., He R., Kiyoko O., Lin S.W. (2017). Berries and other natural products in the pancreatic cancer chemoprevention in human clinical trials. J. Berry Res..

[20] Lee M.Y., Lee N.H., Jung D., Lee J.A., Seo C.S., Lee H., Kim J.H., Shin H.K. (2010). Protective effects of allantoin against ovalbumin (OVA)-induced lung inflammation in a murine model of asthma. Int. Immunopharmacol..

[21] Niu C.S., Chen W., Wu H.T., Cheng K.C., Wen Y.J., Lin K.C., Cheng J.T. (2010). Decrease of plasma glucose by allantoin, an active principle of yam (*Dioscorea* spp.) in streptozotocin-induced diabetic rats. J. Agric. Food Chem..

[22] Chen M.F., Tsai J.T., Chem L.J., Wu T.P., Yang J.J., Yin L.T., Yang Y.L., Chiang T.A., Lu H.L., Wu M.C. (2014). Antihypertensive action of allantoin in animals. Biomed. Res. Int..

[23] Ahn Y.J., Park S.J., Woo H., Lee H.E., Kwon G., Gao Q., Jang D.S., Ryu J.H. (2014). Effects of allantoin on cognitive function and hippocampal neurogenesis. Food Chem. Toxicol..

[24] Song M.Y., Kang S.Y., Oh T.W., Kumar R.V., Jung H.W., Park Y.K. (2015). The Roots of *Atractylodes macrocephala* Koidzumi Enhanced Glucose and Lipid Metabolism in C2C12 Myotubes via Mitochondrial Regulation. Evid. Based Complement. Altern. Med..

[25] Beaudart C., Rizzoli R., Bruyère O., Reginster J.Y., Bive E. (2014). Sarcopenia: Burden and challenges for public health. Arch. Public Health.

[26] Fu Y.C., Ferng L.H.A., Huang P.Y. (2006). Quantitative analysis of allantoin and allantonic acid in yam tuber, mucilage, skin and bulbil of the *Dioscorea* species. Food Chem..

[27] Argilés J.M., Campos N., Lopez-Pedrosa J.M., Rueda R., Rodriguez-Mañas L. (2016). Skeletal Muscle Regulates Metabolism via Interorgan Crosstalk: Roles in Health and Disease. J. Am. Med. Dir. Assoc..

[28] Sayer A.A., Robinson S.M., Patel H.P., Shavlakadze T., Cooper C., Grounds M.D. (2013). New horizons in the pathogenesis, diagnosis and management of sarcopenia. Age Ageing.

[29] Kim J.Y., Kwon Q. (2011). Culinary plant and their potential impact on metabolic overload. Ann. N. Y. Acad. Sci..

[30] Wu Q., Liang X. (2018). Food therapy and medicinal diet therapy of traditional Chinese medicine. Clin. Nutr. Exp..

[31] Komesaroff P.A., Black C.V., Cable V., Sudhir K. (2001). Effects of wild yam extract on menopausal symptoms, lipids and sex hormones in healthy menopausal women. Climacteric.

[32] Dumont N.A., Bentzinger C.F., Sincennes M.C., Rudnicki M.A. (2015). Satellite Cells and Skeletal Muscle Regeneration. Compr. Physiol..

[33] Cole N.J., Hall T.E., Martin C.L., Chapman M.A., Kobiyama A., Nihei Y., Watabe S., Johnston I.A. (2004). Temperature and the expression of myogenic regulatory factors (MRFs) and myosin heavy chain isoforms during embryogenesis in the common carp *Cyprinus carpio* L.. J. Exp. Biol..

[34] Saltiel A.R., Kahn C.R. (2001). Insulin signalling and the regulation of glucose and lipid metabolism. Nature.

[35] Massimiliano G., Francesca G., Jose M., Alvarez S., Mazzoni L., Tamara Y.F.H., Quiles J.L., Bullon P., Battino M. (2016). AMPK as a New Attractive Therapeutic Target for Disease Prevention: The Role of Dietary Compounds AMPK and Disease Prevention. Curr. Drug Targets.

[36] Pistollato F., Battino M. (2014). Role of plant-based diets in the prevention and regression of metabolic syndrome and neurodegenerative diseases. Trends Food Sci. Technol..

[37] Hardie D.G., Ross F.A., Hawley S.A. (2012). AMPK: A nutrient and energy sensor that maintains energy homeostasis. Nat. Rev. Mol. Cell Biol..

[38] Banerjee J., Bruckbauer A., Zemel M.B. (2016). Activation of the AMPK/Sirt1 pathway by a leucine–metformin combination increases insulin sensitivity in skeletal muscle, and stimulates glucose and lipid metabolism and increases life span in *Caenorhabditis elegans*. Metabolism.

[39] Cantó C., Auwerx J. (2009). PGC-1α, SIRT1 and AMPK, an energy sensing network that controls energy expenditure. Curr. Opin. Lipidol..

[40] Taherzadeh-FardE S.C., Akkad D.A., Wieczorek S., Haghikia A., Chan A., Epplen J.T., Arning L. (2011). PGC-1alpha downstream transcription factors NRF-1 and TFAM are genetic modifiers of Huntington disease. Mol. Neurodegener..

[41] Vytla V.S., Ochs R.S. (2013). Metformin increases mitochondrial energy formation in L6 muscle cell culture. J. Biol. Chem..

[42] Kim K.H., Kim M.A., Moon E., Kim S.Y., Choi S.Z., Son M.W., Lee K.R. (2011). Furostanolsaponins from the rhizomes of *Dioscorea japonica* and their effects on NGF induction. Bioorg. Med. Chem. Lett..

[43] Choi K.W., Um S.H., Kwak J.H., Park H.J., Kim K.H., Moon E.Y., Kwon S.T., Pyo S. (2012). Suppression of adhesion molecule expression by phenanthrene-containing extract of bulbils of Chinese Yam in vascular smooth muscle cells through inhibition of MAPK, Akt and NF-κB. Food Chem. Toxicol..

[44] Zhu Q., Zhang Y., Su C., Zie J. (2018). Determination of allantoin and adenosine in *Rhizoma dioscoreae* by HILIC-double wavelength HPLC. Northwest Pharm. J..

[45] Lin K.C., Yeh L.R., Chen L.J., Wen Y.J., Cheng K.C., Cheng J.T. (2012). Plasma glucose-lowing action of allantoin is induced by activation of imidazoline I-2 receptors in streptozotocin-induced diabetic rats. Horm. Metab. Res..

[46] Umegaki H. (2015). Sarcopenia and diabetes: Hyperglycemia is a risk factor for age-associated muscle mass and functional reduction. J. Diabetes Investig..

